# Apoptosis and autophagy induced by pyropheophorbide-α methyl ester-mediated photodynamic therapy in human osteosarcoma MG-63 cells

**DOI:** 10.1007/s10495-016-1243-4

**Published:** 2016-04-23

**Authors:** Qiu Huang, Yun-Sheng Ou, Yong Tao, Hang Yin, Ping-Hua Tu

**Affiliations:** Department of Orthopedics, The First Affiliated Hospital of Chongqing Medical University, No. 1 You Yi Road, Yuanjiagang, Yuzhong District, Chongqing, 400016 China

**Keywords:** Pyropheophorbide-α methyl ester, Photodynamic therapy, Osteosarcoma, Autophagy, Apoptosis

## Abstract

Pyropheophorbide-α methyl ester (MPPa) was a second-generation photosensitizer with many potential applications. Here, we explored the impact of MPPa-mediated photodynamic therapy (MPPa-PDT) on the apoptosis and autophagy of human osteosarcoma (MG-63) cells as well as the relationships between apoptosis and autophagy of the cells, and investigated the related molecular mechanisms. We found that MPPa-PDT demonstrated the ability to inhibit MG-63 cell viability in an MPPa concentration- and light dose-dependent manner, and to induce apoptosis via the mitochondrial apoptosis pathway. Additionally, MPPa-PDT could also induce autophagy of MG-63 cell. Meanwhile, the ROS scavenger *N*-acetyl-l-cysteine (NAC) and the Jnk inhibitor SP600125 were found to inhibit the MPPa-PDT-induced autophagy, and NAC could also inhibit Jnk phosphorylation. Furthermore, pretreatment with the autophagy inhibitor 3-methyladenine or chloroquine showed the potential in reducing the apoptosis rate induced by MPPa-PDT in MG-63 cells. Our results indicated that the mitochondrial pathway was involved in MPPa-PDT-induced apoptosis of MG-63 cells. Meanwhile the ROS-Jnk signaling pathway was involved in MPPa-PDT-induced autophagy, which further promoted the apoptosis in MG-63 cells.

## Background

Osteosarcoma was the most common malignant bone tumor and the age of osteosarcoma patients mainly ranged from 10 to 25 years old. It resulted in a 5-year survival rate of approximately 65–75 % [1]. It was well known that surgery, neoadjuvant chemotherapy and radiotherapy have greatly improved the outcomes of osteosarcoma, but recurrence, metastasis and drug resistance resulted in a poor prognosis of osteosarcoma patients. Moreover, marginal or extended resection for the treatment of osteosarcoma can impair limb function, and radio- and chemotherapy often lead to severe toxicity and side effects. Therefore, novel treatment with strong anti-tumor effects and few side effects was urgently required to improve the prognosis of osteosarcoma patients.

Photodynamic therapy (PDT) was a minimally invasive technology for treating tumors. In PDT, a photosensitizer was systemically administered, and then visible light at a certain wavelength was applied to locally irradiate the tumor [2]. Upon excitation, the photosensitizer reacted with oxygen via the photochemical reaction and photobiological reaction, generating abundant amounts of reactive oxygen species (ROS) which can induce cell death [2]. Therefore, surgery combined with PDT may have some important potential in improving the prognosis of osteosarcoma patients.

Previous studies revealed that PDT can promote the destruction of tumor cells via the following mechanisms: direct cytotoxicity to the tumor cells, induction of anti-tumor immune response, and damage to tumor vessels [3]. Furthermore, it was reported that apoptosis was regarded as one of the main mechanisms mediating PDT-induced tumor cell death [4]. The main morphologic changes in apoptosis (also called type I programmed cell death) included the concentration and fragmentation of chromosomes, cell shrinkage, invagination of cell membrane and generation of apoptosis bodies [5]. In addition, PDT can induce mitochondrial permeability transition and activate the mitochondrial apoptosis pathway [6]. It can also lead to the aggregation of unfolded proteins, leading to endoplasmic reticulum stress and further inducing endoplasmic reticulum-associated apoptosis [7]. Moreover, a caspase-independent pathway was found to mediate the PDT-induced apoptosis [8].

Autophagy, also referred to as type II programmed cell death, is the processes of organelle or protein degradation by the cells themselves [9, 10]. Autophagy and apoptosis were often simultaneously triggered by the same stimulus, but their complicated relationships were controversial. For example, autophagy was found to have the ability to inhibit, delay, or promote the occurrence of apoptosis [11–13]. It was known that PDT showed the potential in inducing autophagy and apoptosis [14], but the detailed mechanisms and the relationships between autophagy and apoptosis remained unclear.

Photosensitizers were regarded as the key factor in PDT. Pyropheophorbide-α methyl ester (MPPa), a derivative of chlorophyll, was known as a second generation photosensitizer. It had many advantages including clear chemical structure, stability, rapid absorption and metabolism and strong photoelectric sensitivity [15]. In this study, we explored the influence of MPPa-PDT on apoptosis and autophagy of human osteosarcoma (MG-63) cells, and investigated the related molecular mechanisms as well as the relationships between apoptosis and autophagy induced by MPPa-PDT.

## Materials and methods

### Reagents and instruments

MG-63 cells was purchased from ATCC (Manassas, USA). MPPa, Monodansylcadaverine (MDC), Hoechst 33258, 2′,7′-dichlorofluorescin diacetate (DCFH-DA), SP600125, 3-methyladenine (3-MA), Chloroquine (CQ) *N*-acetyl-l-cysteine (NAC) were purchased from Sigma-Aldrich (St-Louis, MO, USA). Dulbecco modified Eagle medium (DMEM), Fetal bovine serum (FBS) and trypsin were purchased from Gibco (Carlsbad, CA, USA); JC-1 kits was purchased from Beyotime Biotech (Shanghai, China). Cell viability and cytotoxicity test kits (CCK-8) was purchased from Dojindo Molecular Technologies (Kimamoto, Japan). Annexin V–propidium iodide (PI) double-staining test kit was purchased from Keygen Biotech (Nanjing, China). PDT equipment was purchased from Chongqing Jingyu Laser Technology Co. Ltd. (Chongqing, China).

### Cell culture

MG-63 cells were cultured and sub-cultured in DMEM containing 100 μg/mL penicillin, 100 μg/mL streptomycin, and 10 % FBS (complete medium) at 37 °C in 5 % CO_2_.

### Drug treatment and MPPa-PDT

In the MPPa-PDT group, MG-63 cells in the logarithmic phase were cultured in the dark with different MPPa concentrations (0, 0.25, 0.5, 0.75, and 1.5 μmol/L) for 20 h and then washed three times with phosphate-buffered saline (PBS). The culture medium was replaced, and the cells were exposed to light using an LED with a wavelength of 630 nm in the continuous output mode and a laser power density of 40 mW/cm^2^. The light energy densities were varied by adjusting the exposure time including 0, 30, 60, 120, and 240 s; the corresponding density values were 0, 1.2, 2.4, 4.8, and 9.6 J/cm^2^. Following the light exposure, the cells were cultured in complete culture medium at 37 °C in 5 % CO_2_ in the dark.

In the MPPa-alone group and LED-alone group, cells were subjected to only MPPa treatment or LED light exposure in the same manner compared to the MPPa-PDT group. Before MPPa-PDT treatment, some of the cells were pretreated with 3-MA (5 mmol/L), CQ (10 μmol/L), SP600125 (10 μmol/L), or NAC (10 mmol/L).

### Detection of cell viability by CCK-8 assay

Cells were seeded in 96-well plates at a density of 5 × 10^3^ cells/well with three duplications, and cultured at 37 °C for 24 h. After the attachment, the cells were treated according to the requirement of corresponding groups and further cultured for 24 h. Then, 10 μL CCK-8 was added to each well, and the cells were further incubated for 2 h. A microplate reader was used to detect absorbance at 450 nm. The cell viability was calculated using the following equation:

$${\text{Cell viability }}\left( \% \right){\text{ }} = {\text{ Average OD in study group}}/{\text{average OD in control group }} \times {\text{ 1}}00\;\%$$

where OD was the optical density.

Based on the results of the cell viability assay, we selected an MPPa concentration of 0.75 μM and a light energy density of 4.8 J/cm^2^ as the treatment conditions in the MPPa-PDT group in subsequent experiments.

### Assessment of apoptosis by Hoechst nuclear staining

MG-63 cells were seeded in 24-well plates at a density of 5 × 10^4^ cells/well, treated, and cultured for 3, 6, or 12 h. Then, the culture medium was discarded, and the cells were washed three times with PBS. Next, 10 μg/mL Hoechst 33258 (200 μL) was added to the cells at 37 °C for 5 min in the dark. The cells were observed to evaluate apoptotic changes and photographed with a fluorescence microscope after being washed three times with PBS.

### Assessment of autophagic vacuoles by MDC staining

MG-63 cells were seeded in 24-well plates at a density of 5 × 10^4^ cells/well, treated, and cultured for 3, 6, or 12 h. Then, the culture medium was discarded, and the cells were washed three times with PBS. Next, 0.05 mmol/L MDC (200 μL) was added to the cells at 37 °C for 30 min. Finally, the cells were washed three times with PBS, observed to detect autophagic vacuoles, and photographed with a fluorescence microscope. The fluorescent intensity was analyzed by using Image pro-plus software, version 6.0. Three independent experiments were performed and the representative results were shown.

### Measurement of intracellular ROS level by DCFH-DA staining

MG-63 cells were seeded in 6-well plates at a density of 10 × 10^4^ cells/well, treated, and cultured for 3 h. Then, the culture medium was discarded, and the cells were washed three times with PBS. Next, 10 μmol/L DCFH-DA (1 mL) was added at 37 °C for 20 min. Finally, the cells were observed by fluorescence microscope, and detected by flow cytometry after being digested and collected.

### Assessment of mitochondrial membrane potential by JC-1 staining

MG-63 cells were seeded in 6-well plates at a density of 10 × 10^4^ cells/well, treated, and cultured for 3 h. The culture medium was discarded, and the cells were washed three times with PBS. Next, 1 mL JC-1 working solution and 1 mL complete culture medium was added; the cells were incubated at 37 °C for 20 min, and then washed three times with cold JC-1 staining buffer. Finally, the cells were observed by fluorescence microscope, and detected by flow cytometry after being digested and collected.

### Examination of cell ultrastructure by transmission electron microscopy

MG-63 cells were treated based on the requirement of corresponding groups, collected, and centrifuged at 3, 6, and 12 h after treatment. The cells were then fixed with 2.5 % glutaraldehyde and 1 % osmic acid, dehydrated using gradient ethanol and acetone, embedded, solidified, sliced using an ultramicrotome, stained with 3 % uranium acetate-lead citrate, and observed using transmission electron microscopy (TEM).

### Measurement of apoptosis rate by annexin V–PI double staining and flow cytometry

MG-63 cells were seeded in 6-well plates at a density of 10 × 10^4^ cells/well. All suspended and adherent cells were collected after treatment, and subjected to flow cytometry after annexin V–PI double staining.

### Western blot analysis

Intracellular proteins were extracted after the cell treatments. Protein samples (40 μg) were resolved using sodium dodecyl sulfate polyacrylamide gel electrophoresis and transferred to polyvinylidene fluoride membranes. The membranes were blocked for 1 h at room temperature in Tris-buffered saline containing Tween 20 and 5 % skim milk, and incubated overnight at 4 °C with the primary antibodies (listed and characterized in Table 1). After being washed, the membranes were incubated for 1 h with a secondary antibody labeled with horseradish peroxidase, and developed using electrochemiluminescence. The results were calibrated using β-actin. The gray values of some western bands were analyzed by using Image J software. Three independent experiments were performed and the representative results were shown.

**Table 1:** Primary antibodies used in Western blot analysis

Target	Source	Host	Dilution	Secondary antibody
β-actin	Cell Signaling Technology	Mouse	1:1000	Anti-mouse
Bcl-2	Cell Signaling Technology	Rabbit	1:1000	Anti-rabbit
Total Jnk	Cell Signaling Technology	Mouse	1:1000	Anti-mouse
phospho-Jnk	Cell Signaling Technology	Rabbit	1:1000	Anti-rabbit
Caspase-3	Cell Signaling Technology	Rabbit	1:1000	Anti-rabbit
Cleaved caspase-3	Cell Signaling Technology	Rabbit	1:1000	Anti-rabbit
Beclin-1	Sigma-Aldrich	Rabbit	1:1000	Anti-rabbit
LC-3	Sigma-Aldrich	Rabbit	1:1000	Anti-rabbit
Cytochrome *c*	Abcam	Rabbit	1:1000	Anti-rabbit
Bax	Abcam	Rabbit	1:1000	Anti-rabbit

### Statistical analysis

 Data were represented as mean ± SD and analyzed using SAS. Comparisons among groups were performed using one-way analysis of variance (ANOVA) and two-way ANOVA. Pairwise comparisons within groups were performed using the Student–Newman–Keuls *q* test. P < 0.05 indicated statistical significance.

## Results

### MPPa-PDT decreased MG-63 cell viability

**Fig. 1:**
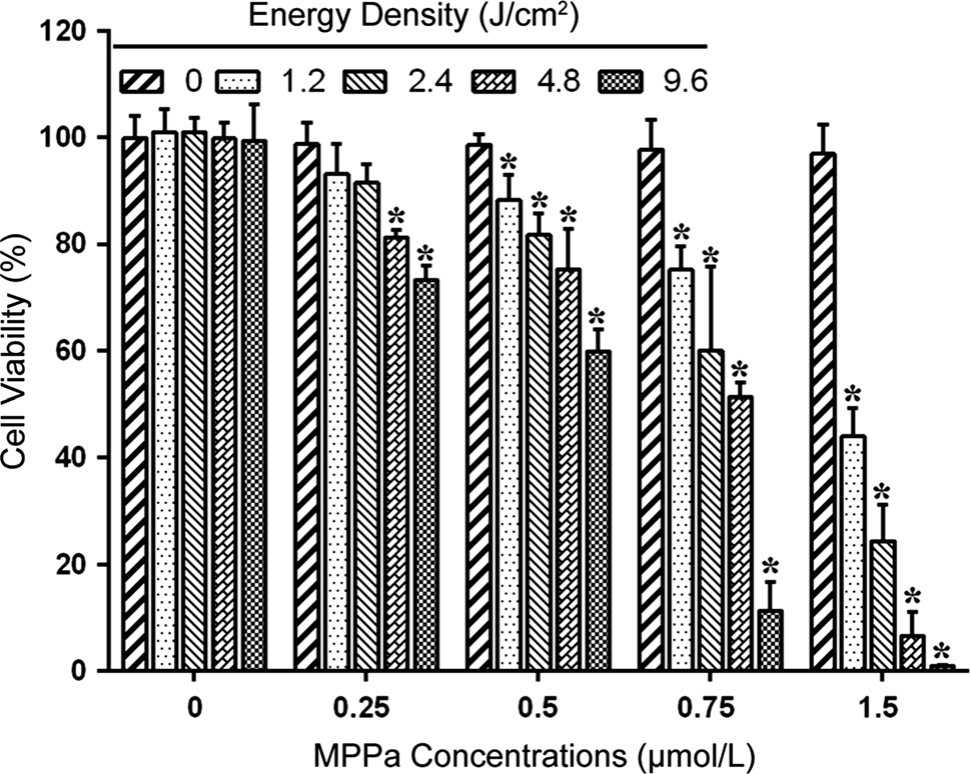
MPPa-PDT decreased MG-63 cell viability. MG-63 cells were treated with different concentrations of MPPa (0, 0.25, 0.5, 0.75, and 1.5 μmol/L) for 20 h, and then irradiated with various light doses (0, 1.2, 2.4, 4.8, and 9.6 J/cm^2^, respectively). At 24 h after irradiation, cell viability was determined using the CCK-8 assay. Datas were presented as mean ± SD from three independent experiments. *P < 0.05 versus the control group

We investigated the influence of the combination of MPPa and LED light exposure (630 nm) on the viability of MG-63 cells (Fig. 1). Compared with the control group (0 μmol/L MPPa, 0 J/cm^2^), the MPPa-alone group and LED-alone group showed no significant inhibition of cell viability (P > 0.05). In the MPPa-PDT group, different MPPa concentrations (0.25, 0.5, 0.75, and 1.5 μmol/L) combined with LED light exposure at different light energy densities (1.2, 2.4, 4.8, and 9.6 J/cm^2^) were used to treat the cells. Cell viability was inhibited in all MPPa-PDT groups, except for those treated with 0.25 μmol/L MPPa combined with 1.2 J/cm^2^ light dose and 0.25 μmol/L MPPa combined with 2.4 J/cm^2^ light dose (P < 0.05). Cell viability was inhibited in an MPPa concentration- and light dose-dependent manner. At a light dose of 4.8 J/cm^2^, the half-maximal inhibitory concentration of MPPa was 0.81 ± 0.02 μmol/L. The inhibition rate in the group that received 0.75 μmol/L MPPa combined with a light dose of 4.8 J/cm^2^ was 48.6 ± 2.71 %. Therefore, we chose an MPPa concentration of 0.75 μmol/L and a light dose of 4.8 J/cm^2^ for the subsequent experiments.

### MPPa-PDT induced apoptosis of MG-63 cells

**Fig. 2:**
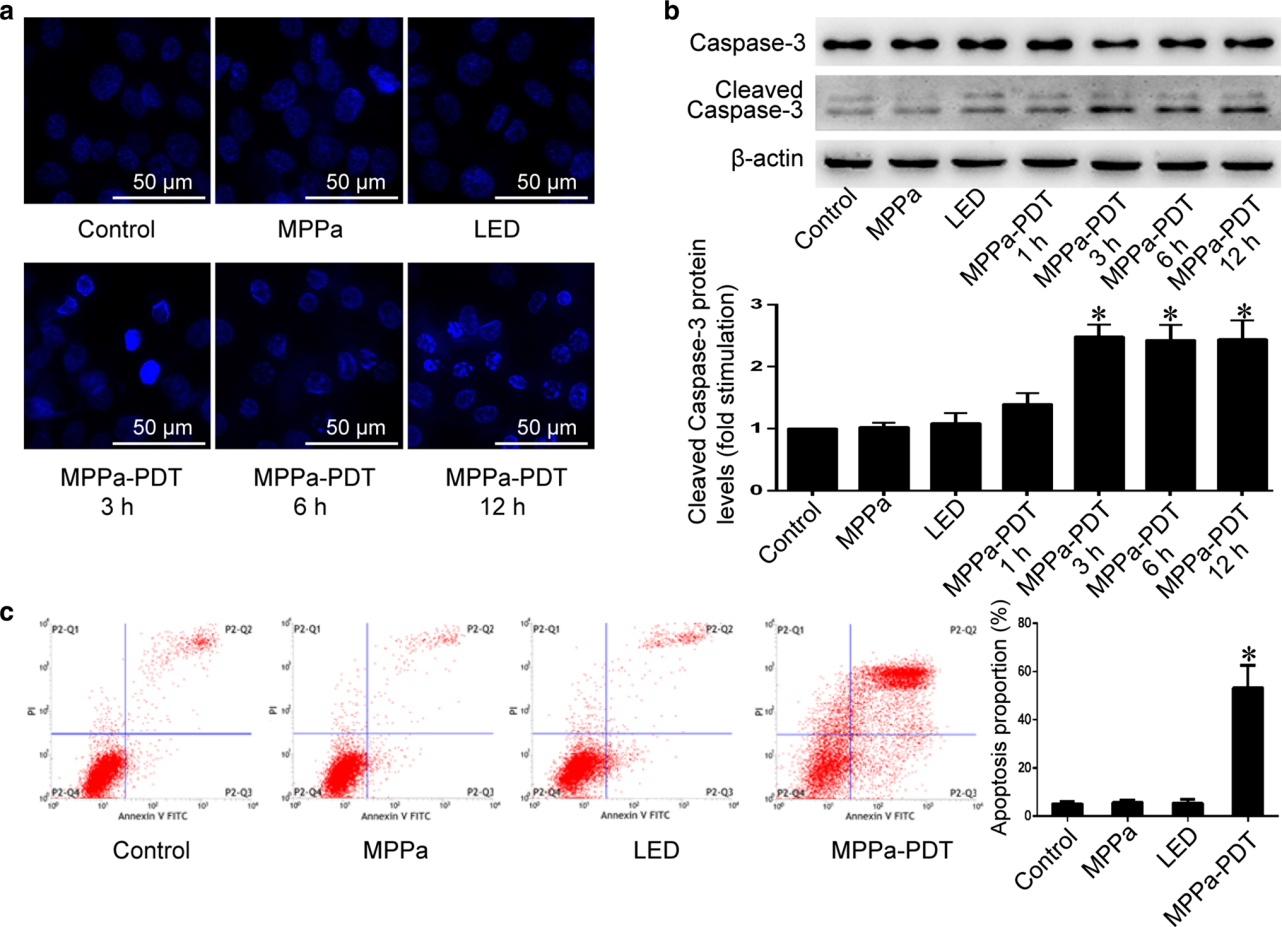
MPPa-PDT induced apoptosis of MG-63 cells. MG-63 cells were treated with MPPa (0.75 μmol/L) for 20 h, and then irradiated with light (4.8 J/cm^2^). **a** At 3, 6, and 12 h after irradiation, apoptotic cells were detected using Hoechst staining (×200). **b** At 1, 3, 6, and 12 h after irradiation, whole-cell lysate was prepared for the assay of cleaved caspase-3 and caspase-3 proteins by Western blotting. Datas were presented as mean ± SD from three independent experiments. *P < 0.05 versus the control group. **c** At 12 h after irradiation, the apoptosis rate was determined using flow cytometric analysis. The apoptosis rate was calculated as the percentage of early apoptotic (annexin V+/PI−) cells plus the percentage of late apoptotic (annexin V+/PI+) cells. Data were presented as mean ± SD from three independent experiments. *P < 0.05 versus the control group

To determine whether MPPa-PDT could induce the apoptosis of MG-63 cells, we used Hoechst 33258 to stain the cell nucleus, and observed the morphological changes of apoptosis by using a fluorescence microscope. At 3, 6, and 12 h after MPPa-PDT treatment, MG-63 cells showed increased chromatin density and appeared bright blue (Fig. 2a). The results also showed the typical morphological changes of apoptosis such as karyopyknosis, condensation, and karyorrhexis. However, no changes occurred in the control group, MPPa-alone group, and LED-alone group. Western blotting revealed the increased expression levels of cleaved caspase-3 at 3, 6, and 12 h after MPPa-PDT treatment compared to that in the other three groups (Fig. 2b).

To quantify the apoptosis level, we performed annexin V–PI staining and flow cytometry. At 12 h after the treatment, there was no significant difference in apoptosis levels among the control, MPPa-alone, and LED-alone groups, but the apoptosis level in the MPPa-PDT group was significantly higher than that in the control group (P < 0.05) (Fig. 2c). These results indicated that MPPa-PDT had the capability to induce the apoptosis of MG-63 cells.

### Mitochondrial pathway was involved in MPPa-PDT-induced apoptosis in MG-63 cells

**Fig. 3:**
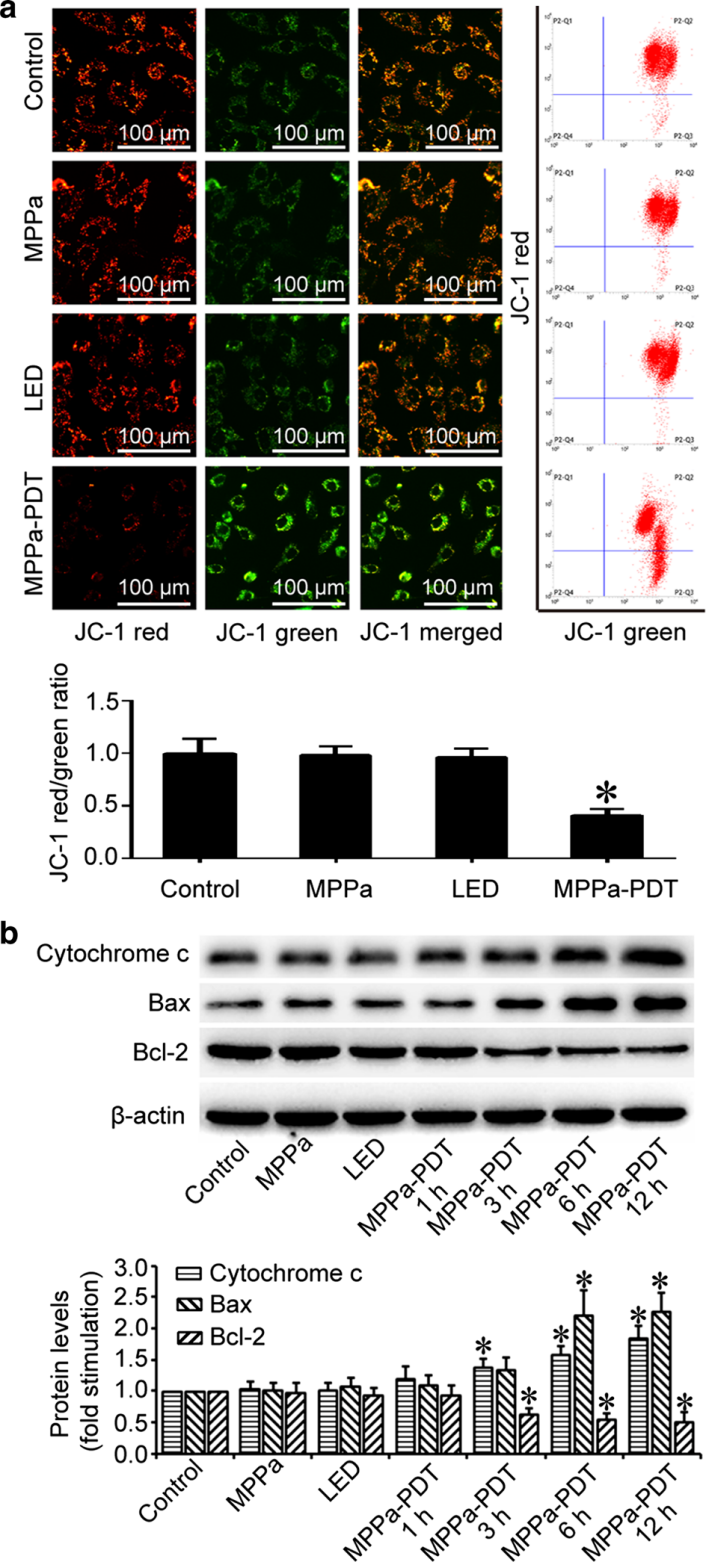
Mitochondrial pathway was involved in MPPa-PDT-induced apoptosis in MG-63 cells. MG-63 cells were treated with MPPa (0.75 μmol/L) for 20 h and then irradiated with light (4.8 J/cm2). **a** At 3 h after irradiation, the mtΔψ was measured using a JC-1 fluorescent probe, fluorescent microscopy (×200), and flow cytometry. The quantitative mtΔψ from each sample was expressed as the ratio of* red* fluorescence intensity over* green* fluorescence intensity. Datas were presented as mean ± SD from three independent experiments. *P < 0.05 versus the control group. **b** At 1, 3, 6, and 12 h after irradiation, whole-cell lysate was prepared for the assay of the Bax and Bcl-2 proteins by Western blotting; cytosol fractions were used to assay cytochrome *c*. Datas were presented as mean ± SD from three independent experiments. *P < 0.05 versus the control group

It was reported that the mitochondrial pathway served as an important mechanism for the induction of apoptosis by PDT, and MPPa was located in the mitochondria [16, 17]. Therefore, we speculated that the mitochondrial pathway was involved in the MPPa-PDT-induced apoptosis of MG-63 cells. JC-1 was a widely used fluorescent probe for detecting mitochondrial membrane potential (MtΔψ). When the membrane potential of the mitochondrion was high, JC-1 aggregated in the mitochondrial matrix, producing JC-1 aggregates and emitting red fluorescence. When the potential was low, JC-1 cannot aggregate and emitted green fluorescence. Thus, the red/green fluorescence ratio indicated the MtΔψ. After MPPa-PDT, the red/green fluorescence ratio of MG-63 cells significantly decreased, as observed by fluorescence microscope and flow cytometry (P < 0.05, Fig. 3a). Moreover, western blotting showed that at 3, 6, and 12 h after MPPa-PDT, the expressions of cytochrome *c* and Bax in the cytoplasm increased, and the expression of Bcl-2 decreased (Fig. 3b). All these results demonstrated the activation of the mitochondrial apoptosis pathway, suggesting that this pathway was involved in the MPPa-PDT-induced apoptosis of MG-63 cells.

### MPPa-PDT induced autophagy of MG-63 cells

**Fig. 4:**
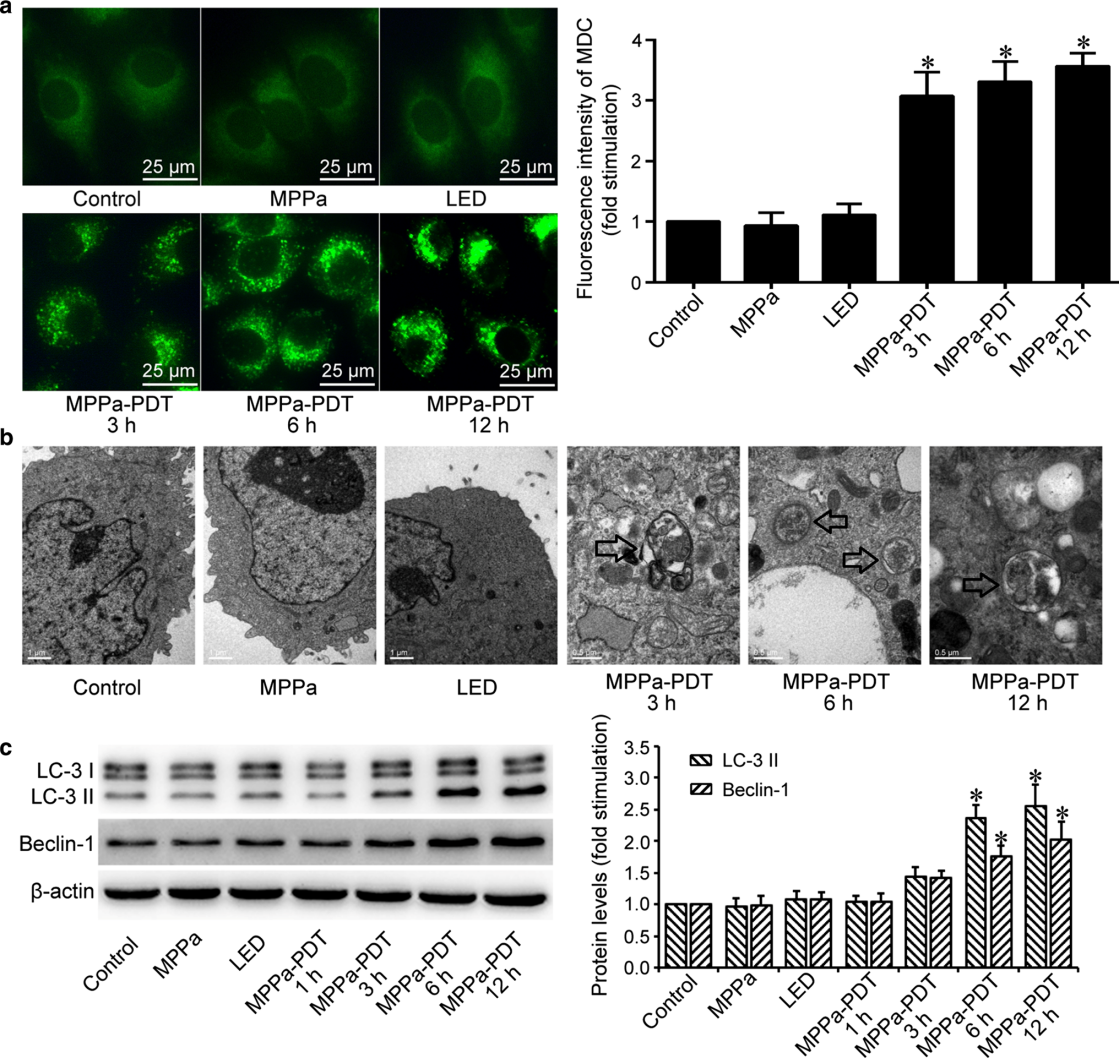
MPPa-PDT induced autophagy of MG-63 cells. MG-63 cells were treated with MPPa (0.75 μmol/L) for 20 h and then irradiated with light (4.8 J/cm^2^). **a** At 3, 6, and 12 h after irradiation, autophagic vacuoles were detected using MDC staining (×400). Data are presented as mean ± SD from three independent experiments. *P < 0.05 versus the control group. **b** At 3, 6, and 12 h after irradiation, autophagosome formation was observed using TEM. The arrows indicated autophagosomes containing intact and degraded cellular debris. **c** At 1, 3, 6, and 12 h after irradiation, whole-cell lysate was prepared for the assay of the LC-3 and Beclin-1 proteins by Western blotting. Datas were presented as mean ± SD from three independent experiments. *P < 0.05 versus the control group

To determine whether MPPa-PDT induced autophagy in MG-63 cells, we used MDC staining and TEM to detect autophagic vacuoles. MDC was regarged as a specific autophagy marker, and it can aggregate in mature autophagic vacuoles (including autophagosomes and autophagic lysosomes) and label them as MDC-positive spots [18]. At 3, 6, and 12 h after treatment, the fluorescent intensity gradually increased, and numerous MDC-positive spots were observed in the MPPa-PDT group (Fig. 4a). However, no such spots was detected in the control group, MPPa-alone group, and LED-alone group, suggesting that MPPa-PDT induced the formation of autophagosomes and autophagic lysosomes. The typical structure of autophagosomes observed by TEM was smooth vacuoles encapsulated by a double layer without ribosomes. Autophagosomes were not observed in the control group, MPPa-alone group, and LED-alone group (Fig. 4b), but were abundant at 3, 6, and 12 h after MPPa-PDT (hollow arrows pointed, Fig. 4b).

The autophagy marker LC-3 had two subtypes: LC-3 I and LC-3 II. Upon the induction of autophagy, LC-3 I was transformed into LC-3 II, which contributed favorably to the formation of autophagosomes. Thus, the expression of LC-3 II indicated the level of autophagy [19]. Beclin-1 played a significant role in inducing autophagy of mammalian cells, and the increase in the level of this protein revealed the initiation of autophagy [20]. In our study, western blotting showed that the level of LC-3 II and Beclin-1 gradually increased at 3, 6, and 12 h after MPPa-PDT (Fig. 4c). These findings suggested that MPPa-PDT could induce the autophagy in MG-63 cells.

### ROS-Jnk signaling pathway was involved in MPPa-PDT-induced autophagy in MG-63 cells

**Fig. 5:**
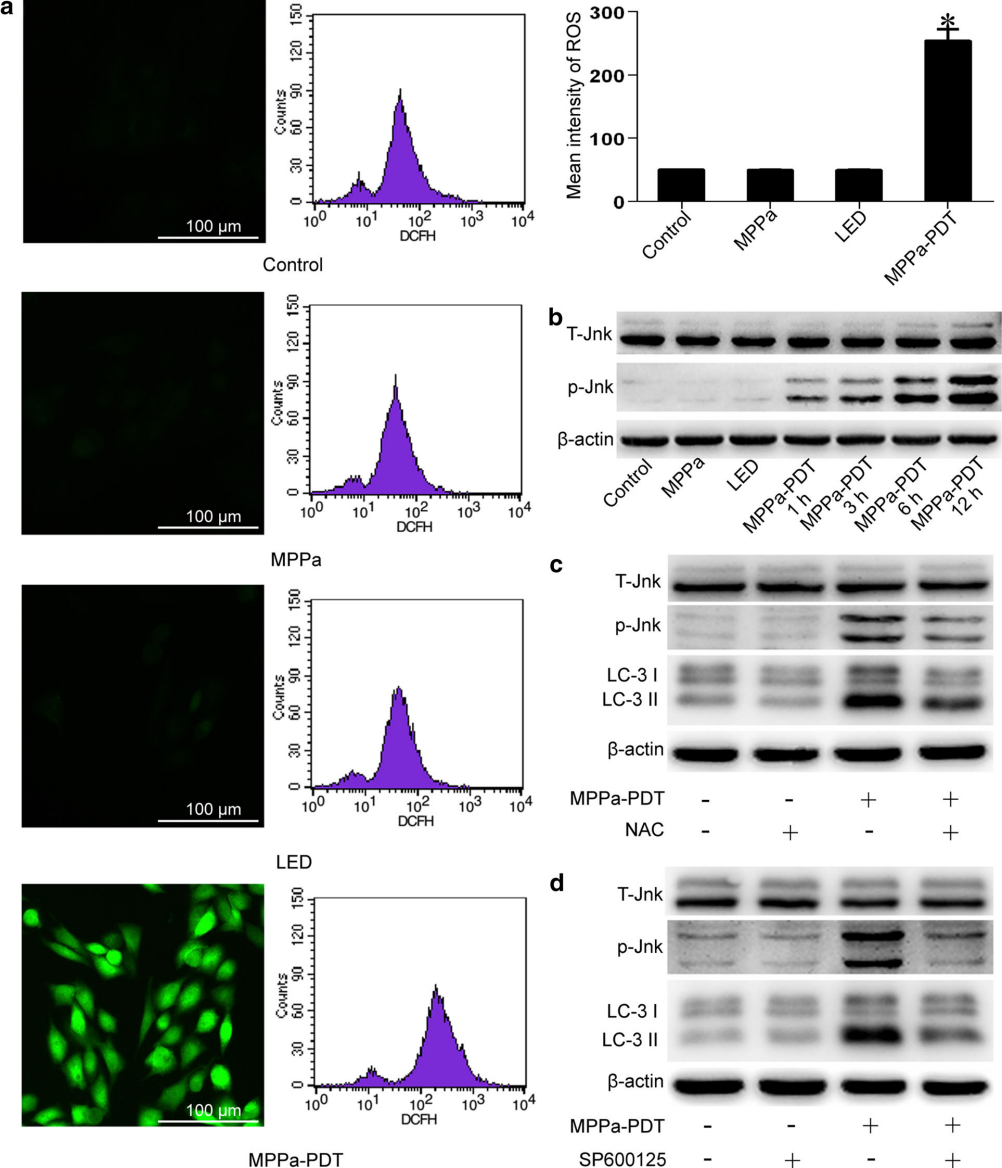
ROS-Jnk signaling pathway was involved in MPPa-PDT-induced autophagy in MG-63 cells. **a** MG-63 cells were treated with MPPa (0.75 μmol/L) for 20 h and then irradiated with light (4.8 J/cm^2^). At 3 h after irradiation, the ROS level was measured using a DCFH-DA fluorescent probe, fluorescent microscopy (×200), and flow cytometry. Representative images were shown. Datas were presented as mean ± SD from three independent experiments. *P < 0.05 versus the control group. **b** MG-63 cells were treated with MPPa (0.75 μmol/L) for 20 h and then irradiated with light (4.8 J/cm^2^). At 1, 3, 6, and 12 h after irradiation, whole-cell lysate was prepared for the assay of phospho-Jnk and total Jnk levels by Western blotting. **c,** **d** MG-63 cells were pretreated with NAC (10 mmol/L) or SP600125 (10 μmol/L) for 1 h in the presence or absence of MPPa-PDT (0.75 μmol/L MPPa+ a light dose of 4.8 J/cm^2^). Some cells received no pretreatment. At 12 h after irradiation, whole-cell lysate was prepared for the assay of the p-Jnk and LC-3 proteins by Western blotting

It was reported that ROS can induce autophagy and activate c-jun NH2-terminal kinase (Jnk). And the Jnk signaling pathway played an important role in the process of autophagy induction [21, 22]. To determine whether the ROS-Jnk signaling pathway was involved in the autophagy in MG-63 cells induced by MPPa-PDT, intracellular ROS levels and Jnk activation were evaluated. We found that the intracellular ROS level was significantly higher in the MPPa-PDT group than that in the control group (P < 0.05, Fig. 5a), as evidenced by fluorescence microscopy and flow cytometry. In contrast, the ROS levels in the MPPa-alone group and LED-alone group showed no significant difference compared to that in the control group (P > 0.05). The level of phosphorylated Jnk gradually increased after MPPa-PDT treatment (Fig. 5b).

We pretreated some cells with a ROS scavenger (NAC, 10 mmol/L) and a Jnk inhibitor (SP600125, 10 μmol/L) for 1 h, and then performed MPPa-PDT. We then measured the levels of phosphorylated Jnk and LC-3 II expression in the pretreated cells. It was found that NAC inhibited Jnk phosphorylation and LC-3 II expression induced by MPPa-PDT (Fig. 5c), suggesting that ROS were involved in the induction of autophagy and activation of Jnk mediated by MPPa-PDT. In the SP600125-treated cells, Jnk inhibition led to the decrease in the autophagy level induced by MPPa-PDT (Fig. 5d). These results suggested that the ROS-Jnk signaling pathway was involved in the MPPa-PDT-induced autophagy in MG-63 cells.

### Relationships between MPPa-PDT-induced autophagy and apoptosis in MG-63 cells

**Fig. 6:**
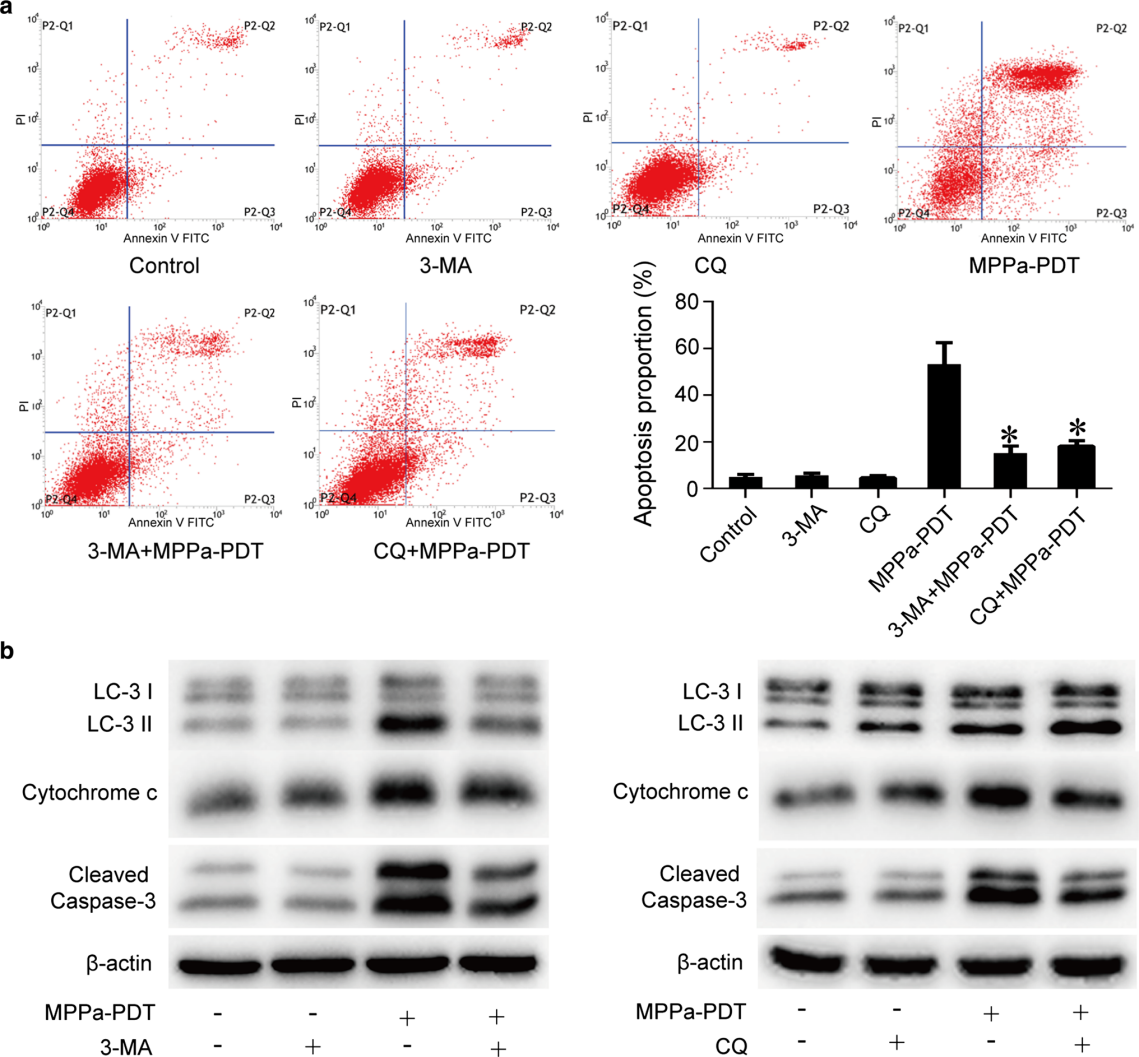
Relationships between MPPa-PDT-induced autophagy and apoptosis in MG-63 cells. MG-63 cells were pretreated with 3-MA (5 mmol/L) and CQ (10 μmol/L) for 1 h in the presence or absence of MPPa-PDT (0.75 μmol/L MPPa+ a light dose of 4.8 J/cm^2^). Some cells received no pretreatment. At 12 h after irradiation, **a** the apoptosis rate was determined using flow cytometric analysis. This rate was calculated using the percentage of early apoptotic (annexin V+/PI−) cells plus the percentage of late apoptotic (annexin V+/PI+) cells. Datas were presented as mean ± SD from three independent experiments. *P < 0.05 versus the MPPa-PDT group. **b** Whole-cell lysate was prepared for the assay of LC-3 and cleaved caspase-3; cytosol fractions were used to assay cytochrome *c* by Western blotting

The relationships between autophagy and apoptosis were complicated, and varied with cell and stimulus types [23]. To determine whether the MPPa-PDT-induced autophagy in MG-63 cells promoted or inhibited apoptosis, we pretreated some cells with 5 mmol/L 3-MA which inhibited autophagy at an early stage [24], and 10 μmol/L CQ which was a late stage autophagy inhibitor [25], and treated them with MPPa-PDT 1 h later. Annexin V–PI staining and flow cytometry were used to evaluate apoptosis. The results revealed that the rate of MPPa-PDT-induced apoptosis was significantly inhibited by 3-MA or CQ pretreatment (P < 0.05, Fig. 6a).

Western blotting was used to detect the influence of 3-MA and CQ pretreatment on cleaved caspase-3 and cytochrome *c* expression induced by MPPa-PDT. Autophagy level was found to significantly decrease after 3-MA or CQ pretreatment, and the expressions of cleaved caspase-3 and cytochrome *c* in the cytoplasm also decreased (Fig. 6b). All the results demonstrated that the inhibition of autophagy in MG-63 cells had the potential in reducing MPPa-PDT-induced mitochondrial apoptosis.

## Discussion

Surgery combined with neoadjuvant chemotherapy currently has become the primary treatment for osteosarcoma [26]. However, it was urgent to develop novel treatments for the patients who can’t bear the toxic and side effects of chemotherapy, and suffer from multi-drug resistance or inoperable tumors caused by vessel and nerve invasion. PDT has been approved by the US Food and Drug Administration for treating tumors [2]. MPPa, a chlorophyll derivative, was a second-generation photosensitizer. MPPa-PDT was reported to exert anti-tumor effects on many types of cancers including colon cancer, nasopharyngeal cancer, lung cancer, osteoclastoma, and cisplatin-resistant human ovarian carcinoma [15, 16, 27–29]. Thus, in this study, we explored the impact of MPPa-PDT on MG-63 cells and the related mechanisms.

Our results showed that MPPa-PDT significantly decreased MG-63 cell viability. After the co-incubation of MG-63 cells with different MPPa concentrations (0–1.5 μmol/L) for 20 h and then exposure to different LED light doses (0–9.6 J/cm^2^), CCK-8 assays showed that treatment with MPPa or light alone did not reduce MG-63 cell viability. In contrast, treatment with MPPa combined with LED light significantly decreased MG-63 cell viability in an MPPa concentration- and light dose-dependent manner. These results indicated that MPPa possessed low dark toxicity and high phototoxicity, making it an ideal photosensitizer.

We next studied the effects of the combination of 0.75 μmol/L MPPa and a light dose of 4.8 J/cm^2^ on MG-63 cells,and found that MPPa-PDT induced apoptosis and autophagy in MG-63 cells. Apoptosis and autophagy are important cell biological processes, which can be induced by the same stimulus, including PDT [3, 30]. The detailed mechanism mediating apoptosis and autophagy were complicated, and differed with photosensitizer type, cellular genotype, and target molecule of the photosensitizer [31]. The clarification of this mechanism is necessary to regulate apoptosis and autophagy, and improve the anti-tumor effects of PDT.

Previous studies showed that the mitochondrion was an important target of PDT. PDT could induce mitochondrial dysfunction and decrease mitochondrial membrane potential, which was followed by the release of cytochrome *c* from the inter-membrane space of the mitochondria into the cytoplasm. Released cytochrome *c* and apoptotic protease-activating factor (Apaf-1) recruited procaspase-9 to form a multimeric protein complex, which led to the autoproteolytic activation of caspase-9 follwed by the activation of downstream caspase-3 to complete the apoptosis process [32, 33]. The Bcl-2 protein family showed some impact on mitochondrial membrane permeabilization, and Bcl-2 can stabilize mitochondrial membrane permeability and prevent cytochrome *c* release. Bax interacted with the channel proteins in the mitochondrial membrane and increased the permeability of the membrane, promoting cytochrome *c* release from the mitochondria [34]. The mitochondrial pathway played a very important role in the MPPa-PDT-induced apoptosis of prostate cancer and ovarian cancer cells [29, 35]. In this study, we found that the intracellular mitochondrial membrane potential was significantly decreased at 3 h after MPPa-PDT, as determined by JC-1 staining. Furthermore, western blotting showed that MPPa-PDT induced cytochrome *c* release, decreased Bcl-2 expression, and increased Bax expression in MG-63 cells. All these results suggested that the mitochondrial signaling pathway played an important role in MPPa-PDT-induced autophagy in MG-63 cells.

ROS served as the basic mechanism mediating PDT-induced cell death and cell autophagy. ROS can directly induce autophagy by upregulating autophagy-associated gene (ATG) expression or indirectly promote autophagy by activating the Jnk signaling pathway [36–38]. Activated Jnk can upregulate the expression of Atg5, Atg6 and Atg7 via c-jun phosphorylation to induce autophagy [38]. Furthermore, the activation of Jnk resulted in the phosphorylation of Bcl-2 which upregulated free Beclin-1(Atg6) levels by releasing Beclin-1 from the Beclin-1/Bcl-2 complex, thereby promoting autophagy [39].

In our study, ROS and Jnk phosphorylation significantly increased after MPPa-PDT; the Jnk inhibitor, SP600125, significantly inhibited the induction of autophagy by MPPa-PDT. These indicated that the Jnk signaling pathway was involved in the induction of autophagy by MPPa-PDT in MG-63 cells. Through exploring the upstream signaling of Jnk, we found that NAC significantly decreased MPPa-PDT-induced Jnk phosphorylation and autophagy, suggesting that ROS played an important role in inducing autophagy and activating Jnk. All these results indicated that the ROS-Jnk signaling pathway was involved in the induction of autophagy in MG-63 cells by MPPa-PDT.

Thus, we proved that MPPa-PDT could induce apoptosis and autophagy in MG-63 cells, but the relationships between them were still unclear. Wei et al. [12] found that the inhibition of autophagy using pharmacological inhibitors and Atg5 gene silencing significantly increased PDT-induced apoptosis in PROM1/CD133^+^ colon cancer cells, suggesting that autophagy had an anti-apoptotic effect. Ji et al. [31] found that the death of PC12 and CL1-0 cells induced by 5-aminolevulinic acid-PDT could be inhibited by an autophagy inhibitor, rather than a caspase inhibitor, indicating that the cell death was autophagic in nature. In our study, 3-MA and CQ pretreatment decreased MPPa-PDT-induced apoptosis in MG-63 cells, revealing that the autophagy induced by MPPa-PDT promoted MG-63 cell apoptosis. These results were consistent with the findings of Wang et al. [40], who reported that the inhibition of autophagy decreased YM155-induced apoptosis in prostate cancer cells, indicating that the apoptosis was autophagy-dependent. These indicated that autophagy can inhibit, delay or promote apoptosis. And the redox state of cells was regarded as a key factor to regulate apoptosis, autophagy and their relationships [41]. Factors such as photosensitizer characteristics, cell line type, and photodynamic dose could influence the balance between the formation and degradation of intracellular ROS, and further influence the cellular redox state [11, 42, 43]. Therefore, the relationship between autophagy and apoptosis induced by PDT might be related to the above factors. Clarifying the relationship between the autophagy and apoptosis induced by MPPa-PDT may help regulate these processes and improve the anti-tumor effects of MPPa-PDT.

## Conclusion

In this study, we found that the mitochondrial apoptosis pathway was involved in MPPa-PDT-induced apoptosis in MG-63 cells, while the ROS-Jnk signaling pathway was involved in MPPa-PDT-induced autophagy, which in turn further promoted apoptosis in MG-63 cells. These results will enrich our understanding of the mechanisms mediating PDT-induced tumor cell death and the relationships between autophagy and apoptosis. Further investigation is required to determine how to regulate autophagy and apoptosis in order to improve the anti-tumor effects of MPPa-PDT.

## Abbreviations

MPPa: Pyropheophorbide-α methyl ester
PDT: Photodynamic therapy
Jnk: c-jun NH2-terminal kinase
ROS: Reactive oxygen species
NAC: *N*-Acetyl-l-cysteine
MDC: Monodansylcadaverine
3-MA: 3-Methyladenine
CQ: Chloroquine
MtΔψ: Mitochondrial membrane potential
DCFH-DA: 2′,7′-Dichlorodihydrofluorescein diacetate
JC-1: 5,5′,6,6′-Tetrachloro-1,1′,3,3′-tetraethyl-imidacarbocyanine iodide
